# Orthogeriatric Fracture Syndrome: Time Is Mobility!

**DOI:** 10.3390/jcm15166349

**Published:** 2026-08-17

**Authors:** Johannes Dominik Bastian

**Affiliations:** Department of Orthopaedic Surgery and Traumatology, Inselspital, Bern University Hospital, University of Bern, 3010 Bern, Switzerland; johannes.bastian@insel.ch

## 1. From an Event to a Syndrome

**Figure 1:**
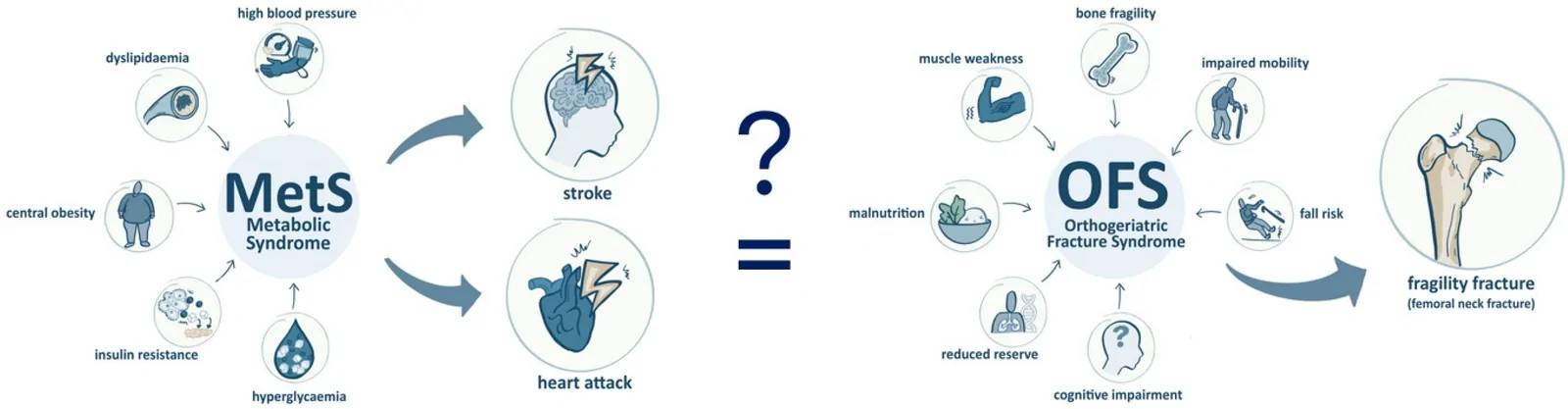
MetS and OFS: syndrome-based thinking as a key to prevention and successful outcomes; established for Metabolic Syndrome (MetS), but it is yet to be evaluated for the proposed Orthogeriatric Fracture Syndrome (OFS). Equivalence remains unproven, as symbolized by the question mark above the equals sign. Reproduced from (contribution 1).

Bissoto et al. propose the Orthogeriatric Fracture Syndrome (OFS) as a unifying framework for the multidimensional vulnerability from which fragility fractures emerge (contribution 1). Their text-mining analysis of 26 million PubMed abstracts identified a structured, non-random network connecting osteoporosis, sarcopenia, malnutrition, impaired gait, falls, frailty, cognition, old age, and fracture outcomes. The fragility fracture can therefore be understood not simply as an isolated skeletal injury, but as the visible endpoint of an interacting and potentially modifiable risk constellation (Figure 1).

Syndrome-based thinking, with a syndrome being defined as the coexistence of multiple characteristic clinical features [1,2], matters because it makes distributed risks clinically visible. Earlier recognition can trigger earlier management, translate fragmented evidence into best-practice pathways, preserve mobility and extend healthy, independent years. It may also shift expenditure from repeated acute repair and dependency towards prevention and coordinated, potentially cost-effective care. OFS is not yet a validated diagnosis: its domains, thresholds, predictive value, and responsiveness to intervention require prospective validation.

## 2. Learning from the Metabolic Syndrome: Time Is Mobility

The metabolic syndrome brought obesity, hypertension, dyslipidemia, and impaired glucose regulation together as a recognizable precursor of myocardial infarction or stroke [3,4]. Its value was not merely a label, but improved risk recognition and preventive action before irreversible organ damage. Once cardiovascular events occur, urgency is captured by “time is muscle (heart attack)” and “time is brain (stroke)”. For OFS, the corresponding principle is “time is mobility (fragility fracture)”. Delay in recognizing vulnerability, diagnosing and stabilizing the fracture, initiating orthogeriatric co-management, mobilizing, rehabilitating, and preventing the next fracture can result in irreversible functional decline. Accumulated delays may cost independence (mobility).

## 3. Seven Domains for Recognition and Action

A pragmatic OFS framework might comprise seven domains: (1) bone strength—osteoporosis, previous fragility fracture, or elevated validated fracture risk [5,6,7]; (2) muscle function—reduced strength or muscle mass and sarcopenia [8,9,10]; (3) nutrition—malnutrition defined by established criteria [11]; (4) mobility—impaired gait, balance, or dependence on walking aids [12,13,14]; (5) fall risk—previous or recurrent falls, fear of falling, polypharmacy, sensory or environmental risks [15,16]; (6) physiological reserve—frailty measured with validated instruments [17,18,19,20,21]; and (7) cognition—mild cognitive impairment or dementia identified clinically or by validated testing [22,23,24,25]. A threshold such as impairment in four of seven domains is hypothesis-generating, but it is not yet a diagnostic criterion.

## 4. What This Special Issue Adds

The contributions to this Special Issue can be understood along the patient journey rather than as disciplinary silos:

First, biological vulnerability extends beyond bone mineral density. The studies identify vitamin D deficiency and impaired absorption, malnutrition, sarcopenia, frailty, atypical femoral fracture pathways (even without bisphosphonate exposure), and chronic kidney disease–mineral and bone disorder (CKD-MBD) as clinically relevant and partly modifiable determinants of fracture risk and recovery. Point-of-care Ultrasound (POCUS) muscle assessment and targeted metabolic and nutritional evaluation may facilitate active case finding, although their prognostic and therapeutic value requires further validation (contribution 2–7).

Second, the fracture phenotype defines the immediate mechanical problem but not the whole patient. Across pelvic, intertrochanteric, native and periprosthetic acetabular, femoral-neck, and peri-implant fractures, treatment should integrate fracture morphology, bone quality, implant status, comorbidity, functional demand, and physiological reserve. The shared objectives are stable reconstruction, safe early mobilization, and preservation of independence. Outcomes should therefore include pain, mobility, return to the previous living situation, function, complications, reoperation, and patient satisfaction—not radiographic success alone. Contemporary fixation and arthroplasty strategies show promising results, but comparative evidence and standardized treatment and rehabilitation pathways remain limited (contribution 8–15).

Third, risk stratification should guide care and recovery rather than merely predict mortality. The studies support combining validated mortality models with chronological age, comorbidity, frailty, premorbid functional capacity, intraoperative physiology (particularly lactate) and immediate postoperative care requirements such as post-anesthesia care unit (PACU) overnight stay. Admission electrocardiographic (ECG) findings, particularly atrial fibrillation when considered alongside head trauma and cerebrovascular disease, may provide additional early prognostic information. Mortality models require local validation and recalibration, while frailty and functional measures add information not captured by age or conventional perioperative scores alone (contribution 16–19).

Fourth, technical success depends on restoring mechanical stability while limiting physiological burden. Anatomical reduction, appropriate implant positioning, controlled compression, stable fixation, and constructs capable of supporting early loading are recurring principles. Biomechanical equivalence under moderate loading does not necessarily imply equal stability at higher loads, while complex revision procedures require particular attention to complication risk and cumulative physiological stress in older adults (contribution 20–22).

Fifth, weight-bearing is not equivalent to recovery. Rehabilitation must connect mechanical stability with actual mobility, function, and independence. Weight-bearing prescriptions are inconsistently standardized and frequently difficult for older patients to follow; a single session of audio biofeedback appears insufficient to ensure adherence. Recovery assessment should therefore combine prescribed loading with objective mobility, muscle and nutritional status, pain, living situation, and patient-reported function (contribution 3, 4, 7, 14, 15, 23).

Finally, “time is bone” highlights persistent gaps in secondary fracture prevention. Fracture Liaison Services should rapidly identify patients who can begin treatment without delay and reserve DXA for cases in which the result is likely to alter the treatment decision. Effective secondary prevention should integrate osteoporosis treatment with the evaluation of vitamin D status, nutrition, sarcopenia, renal and metabolic abnormalities, falls risk, adherence, and structured follow-up (contribution 2–7, 24, 25).

## 5. From Awareness to Best Practice

The next step is to validate a feasible OFS core set, determine which domain combinations predict fracture, functional decline, and recovery, and test whether OFS-guided pathways improve outcomes beyond established orthogeriatric co-management. Research should prioritize mobility, return home, activities of daily living, delirium, refracture, quality of life, and healthy years—not mortality alone. Registers and reimbursement should reward coordinated prevention, perioperative care, rehabilitation, and secondary fracture prevention.

OFS offers a shared language for orthopedic surgeons, geriatricians, anesthetists, nurses, nutrition and rehabilitation specialists, bone-health services, and primary care. Its promise is earlier risk recognition, earlier management, translation into best-practice guidance, preservation of healthy independent years, and a cost-effective shift from repeated repair to prevention. In myocardial infarction, time is muscle; in stroke, time is brain. In the Orthogeriatric Fracture Syndrome, time is mobility—and mobility is a prerequisite for independence.
